# Comparison of assembly algorithms for improving rate of metatranscriptomic functional annotation

**DOI:** 10.1186/2049-2618-2-39

**Published:** 2014-10-28

**Authors:** Albi Celaj, Janet Markle, Jayne Danska, John Parkinson

**Affiliations:** 1 Molecular Structure and Function, Hospital for Sick Children, Peter Gilgan Center for Research and Learning, 686 Bay Street, Toronto, Ontario M5G 0A4, Canada; 2 Department of Molecular Genetics, University of Toronto, Toronto, Ontario M5S 3E1, Canada; 3 Department of Immunology, University of Toronto, Medical Sciences Building, 1 King’s College Circle, Room 5207, Toronto, Ontario M5S 1A8, Canada; 4 Genetics and Genomic Biology, Hospital for Sick Children, Peter Gilgan Center for Research and Learning, 686 Bay Street, Toronto, Ontario M5G 0A4, Canada; 5 Department of Biochemistry, University of Toronto, Toronto, Ontario M5S 1A8, Canada; 6 Current address: Laboratory of Human Genetics of Infectious Diseases, Rockefeller University, New York, NY 10065, USA

**Keywords:** Microbiome, Metatranscriptomics, Sequence assembly, Bioinformatics, RNA sequencing

## Abstract

**Background:**

Microbiome-wide gene expression profiling through high-throughput RNA sequencing (‘metatranscriptomics’) offers a powerful means to functionally interrogate complex microbial communities. Key to successful exploitation of these datasets is the ability to confidently match relatively short sequence reads to known bacterial transcripts. In the absence of reference genomes, such annotation efforts may be enhanced by assembling reads into longer contiguous sequences (‘contigs’), prior to database search strategies. Since reads from homologous transcripts may derive from several species, represented at different abundance levels, it is not clear how well current assembly pipelines perform for metatranscriptomic datasets. Here we evaluate the performance of four currently employed assemblers including *de novo* transcriptome assemblers - Trinity and Oases; the metagenomic assembler - Metavelvet; and the recently developed metatranscriptomic assembler IDBA-MT.

**Results:**

We evaluated the performance of the assemblers on a previously published dataset of single-end RNA sequence reads derived from the large intestine of an inbred non-obese diabetic mouse model of type 1 diabetes. We found that Trinity performed best as judged by contigs assembled, reads assigned to contigs, and number of reads that could be annotated to a known bacterial transcript. Only 15.5% of RNA sequence reads could be annotated to a known transcript in contrast to 50.3% with Trinity assembly. Paired-end reads generated from the same mouse samples resulted in modest performance gains. A database search estimated that the assemblies are unlikely to erroneously merge multiple unrelated genes sharing a region of similarity (<2% of contigs). A simulated dataset based on ten species confirmed these findings. A more complex simulated dataset based on 72 species found that greater assembly errors were introduced than is expected by sequencing quality. Through the detailed evaluation of assembly performance, the insights provided by this study will help drive the design of future metatranscriptomic analyses.

**Conclusion:**

Assembly of metatranscriptome datasets greatly improved read annotation. Of the four assemblers evaluated, Trinity provided the best performance. For more complex datasets, reads generated from transcripts sharing considerable sequence similarity can be a source of significant assembly error, suggesting a need to collate reads on the basis of common taxonomic origin prior to assembly.

## Background

Innovations in culture-independent microbiology, coupled with rapid advances in high-throughput sequencing (HTS), are beginning to profoundly transform our understanding of the relationships between microbial communities and their environments. For example, it is becoming increasingly clear that the composition of the human gut microbiome plays a major role in the development of many human diseases including obesity, type 1 diabetes, inflammatory bowel disease, and autism [1-7]. To date, studies on the human microbiome have largely focused on the use of 16S rRNA surveys which examine shifts in the composition of microbial communities [8-10]. However, such studies lend only limited insight into microbiome function. Recently, we and others have pioneered the use of microbiome-wide gene expression profiling via RNA sequencing (RNA-Seq) or ‘metatranscriptomics’ as a route to functionally interrogate a microbiome [11-14]. Key to exploiting the full potential of these datasets is the ability to accurately assign and annotate sequence reads to known transcripts [12], a challenge that is complicated by the inherent complexity associated with microbial communities as well as the lack of a comprehensive set of reference genomes.

In typical RNA-Seq applications, sequence reads are mapped onto a reference genome to yield expression profiles for each gene. In the absence of reference genomes, sequence annotation is typically performed through sequence similarity searches against databases of previously annotated genes or proteins [15,16]. However, for sequencing technologies capable of generating the hundreds of millions of reads required for metatranscriptomic analyses, resultant read lengths tend to be short (e.g., 75–150 bp), reducing our ability to identify meaningful sequence matches with confidence. Since many reads may derive from the same transcript, assembling reads into longer contiguous sequences (‘contigs’) offers a useful avenue for improving read annotation. However, unlike reads generated from a single organism, RNA-Seq analysis of complex microbial communities poses the additional complication that the multiple species may be represented at significantly different levels of abundance. To date, several tools, based on the use of de Bruijn graphs, have been developed to assemble sequence data *de novo*: Metavelvet [17] was originally developed to assemble metagenomic datasets, while Oases [18] and Trinity [19] were developed to specifically assemble RNA sequence data. More recently, a dedicated metatranscriptomics assembler has also been described that relies on the use of paired-end reads [20]. Due to the absence of suitable datasets, it is not clear how assemblers, previously developed for assembling other types of sequence data, compare with a dedicated tool for assembling metatranscriptomic datasets and, furthermore, what types of error each may introduce.

One potential source of error in transcript assembly is the incorporation of reads from several similar transcripts such as members of the same gene family or the merging of orthologs from different species. While such errors may impact taxonomic assignments, they may have minimal impact on functional assignments. A more serious source of error is that unrelated transcripts sharing a region of high sequence similarity but distinct abundance and/or function may be merged into a single erroneous contig. In such cases, the expression value of the rarer transcript can be masked by the more abundant transcript and/or, depending on the annotation pipeline, only a single function may be ascribed. Our aim was to undertake a systematic evaluation of current assembly tools across multiple metatranscriptomic datasets to assess their performance and determine if the incidence of contig reconstruction errors is likely to impact downstream analyses.

We focused on a metatranscriptomic data from previous 76-bp single-end RNA sequence reads, as well as a new data set of 76-bp paired-end reads, from a microbial consortium isolated from the large intestine (cecum) of inbred non-obese diabetic (NOD) mice, a model of spontaneous type 1 diabetes [12]. Here we show how different approaches to sequence assembly impacts transcript annotation and how complex datasets may be more prone to annotation error.

## Results and discussion

### Assembly significantly improves the number of annotated reads

**Figure 1:**
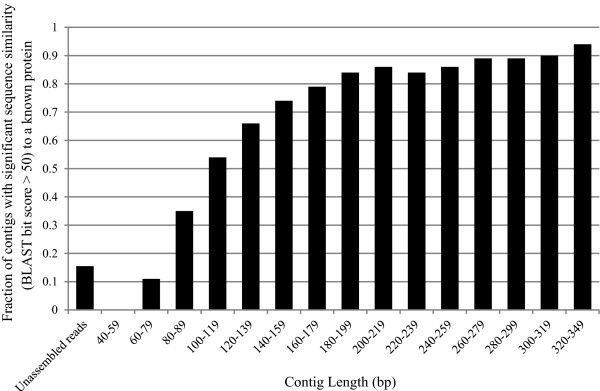
**Trinity-based assembly of short-read metatranscriptomic data improves annotation.** The *de novo* transcriptome assembler, Trinity [19], was applied to a metatranscriptomic dataset generated from a non-obese diabetic (NOD) mouse cecal sample (NOD503CecMN). The probability of obtaining a significant sequence alignment (bit score >50) to a known protein increases with contig length. Contigs greater than 79 bp demonstrate greater annotation potential compared to unassembled reads.

Assembly of next-generation sequencing reads promises to improve automated annotation through sequence similarity searches by improving sequence length and reducing read errors [21]. We were interested in examining whether these approaches were useful for metatranscriptome datasets. To examine how assembly impacts annotation of putative transcripts through sequence similarity searches, we first applied the Trinity assembly algorithm to a previously published dataset of 516,881 76-bp single-end reads of putative bacterial mRNA origin obtained from a NOD mouse cecal sample [12] (designated NOD503CecMN; see ‘Methods’ and Additional file 1). Using default parameters with a 51-bp minimum contig size (here we include reads with at least 50 high-quality base calls), 78.9% of the reads could be assembled into 48,469 contigs varying in length from 51 to 1,317 bp. For the unassembled reads, only 15.5% of the unassembled reads had significant sequence similarity (bit score >50) to a known bacterial protein as determined through BLAST [22] (Figure 1). We obtained similar results for 11 additional datasets generated from related NOD mouse intestinal samples (see ‘Methods’ and Additional file 2). Considering the assembled reads, the proportion of contigs with a significant sequence similarity match to a known gene (‘annotatable contigs’) increased with contig length (Figure 1). The relationship appears asymptotic with 80%–90% of contigs with lengths in excess of 200 having significant sequence similarity matches to known proteins. This finding is consistent with previous reports that ~10% of genes from newly sequenced bacterial genomes appear novel (i.e., lack significant sequence similarity to an existing gene) [23]. On the other hand, for reads which could not be aligned to a contig created by Trinity, only 9.8% had significant sequence similarity (bit score >50) to a known bacterial protein. With read lengths from the Illumina HiSeq sequencing platform beginning to approach 250 bp, we expect that attention for assembly algorithms will focus more on the quality of annotation, rather than simply obtaining an annotation. In the next section, we explore the performance of several algorithms to assemble metatranscriptomic read data.

### Comparison of assembly algorithms on single-end sequence data

**Figure 2:**
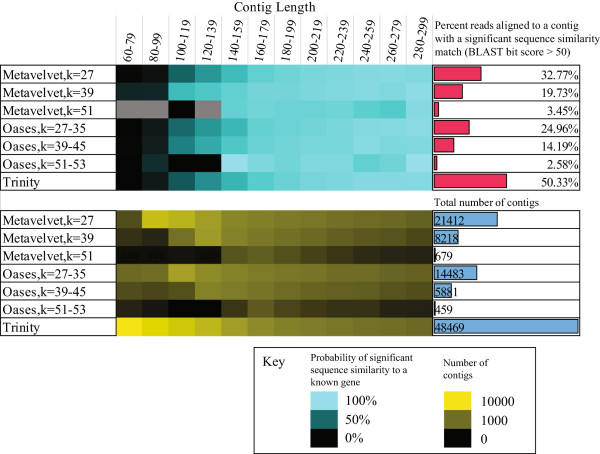
**Performance of three short-read assemblers on a single-end metatranscriptomic dataset.** Three different single-end assemblers (with varying *k*-mer parameters where appropriate) were applied to the NOD503CecMN single-end dataset and evaluated on the basis of: 1) the probability of contigs of different lengths having significant sequence similarity (bit score >50) to a known protein, as well as the percentage of reads which could be annotated (top panel), and 2) contig length distributions (bottom panel). While the assemblers varied greatly in the contig length distribution, number of contigs assembled, and number of reads which could be matched to an annotated contig, all contigs over 180 bp, irrespective of the assembler used to generate them, had a consistently high probability of having significant sequence similarity to a known protein.

**Figure 3:**
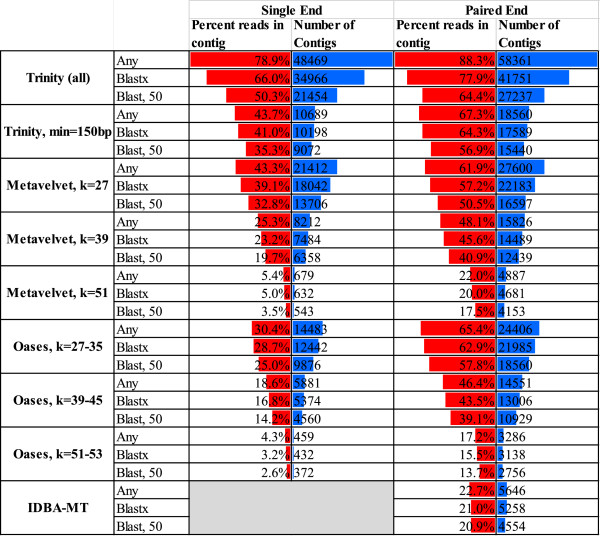
**Performance of four short-read assemblers on both single- and paired-end metatranscriptomic datasets.** Assembly performance was assessed using both single-end and paired-end datasets generated from the NOD503CecMN sample. Comparisons between the two datasets are presented for each assembler/parameter combination except IDBA-MT which requires paired-end data. Assemblers were evaluated on the basis of: 1) number of contigs assembled, 2) percentage of reads that map to assembled contigs, and 3) whether contigs have sequence similarity to a known protein at two levels of stringency.

Given that increasing length improves our ability to annotate, we were interested in identifying the assembler that both maximizes contig length as well as the number of reads incorporated into contigs. We applied three established algorithms, Metavelvet [17], Oases [18], and Trinity [19] to our dataset of 516,881 single-end reads of putative bacterial mRNA origin. For Metavelvet and Oases, we tested a range of *k*-mer values to examine the impact of low (*k* = 27), medium (*k* = 39), and large (*k* = 51) word size on assembly. As Oases combines multiple *k* values into a single assembly, we increased the upper limit of the *k*-mer parameter until less than 5% additional contigs were generated, consistent with author guidelines [18]. For all three assemblers, we obtained similar relationships between contig length and probability of obtaining a significant sequence similarity match to a known gene. Contigs of length 180–200 bp had probabilities of obtaining a significant sequence similarity match ranging from 79% to 83% depending upon the method of assembly (Figure 2). However, the number of reads that could be assembled, as well as the number of contigs, varied between the three algorithms. Trinity provided the best performance in terms of reads assembled into an annotatable contig (as defined through possessing a BLASTX bit score >50 to a known transcript, Figure 3) and total number of annotatable contigs (21,454 vs. 13,706 for the next best-performing algorithm, Metavelvet with *k* = 27). Of these, only 5,561 contigs were >180 bp in length compared to 3,856 for Metavelvet (*k* = 27). Furthermore, Trinity assembled contigs had a lower N50 value than those generated with Metavelvet (*k* = 27) (130 vs. 156 bp, respectively). While this might suggest that contigs assembled with Trinity may impact annotation performance, we found that 50.3% of the 516,881 reads aligned to a Trinity assembled contig that could be annotated (compared to 32.8% Metavelvet with *k* = 27) despite a similar minimum contig size (51 bp for Trinity, 54 bp for Metavelvet). Notably, increasing the minimum contig size to 150 bp for Trinity still resulted in 35.3% of reads mapping to an annotatable contig. For both Metavelvet and Oases, word size (*k*) had a significant impact on performance, with higher values resulting in a low number of reads assembling into annotatable contigs. This latter finding appears to contradict the recommendation to use a *k*-mer length greater than 51 for reads longer than 65 bp for Metavelvet assemblies (http://metavelvet.dna.bio.keio.ac.jp/) and may reflect a greater emphasis on reconstruction accuracy, rather than annotation performance. Alternatively, these differences may arise from inherent sequence differences between metagenomic and metatranscriptomic datasets. For example, compared to metagenomic samples, transcript abundances in metatranscriptome samples are influenced not only by taxonomic representation but also their relative expression, and subject to more error-prone RNA sample preparation processes [24].

Given the superior performance of Trinity over the other methods, we next explored the overlap between assemblies to determine the agreement between the different assembly solutions. We defined the overlap between two datasets, d1 and d2 (where d1 has fewer reads assembled into contigs than d2), as the percentage of reads in d1 assembled into contigs that were also assembled into contigs in d2 (see ‘Methods’). For all assembled contigs, as well as annotatable contigs, we observed a high degree of overlap in assembled reads, suggesting that each algorithm does not assemble a unique fraction of reads (Table 1, Additional file 3). Perhaps not surprisingly, the Trinity-based assembly had the largest overlap with the other assemblies, placing 89%–97% of assembled reads into annotatable contigs (Table 1). Due to the high overlap between assemblies, we did not explore combining results from different algorithms. Moreover, merging of multiple assembly results has been reported to result in additional errors, at least when applied to Roche 454 sequencing data [25].

**Table 1:** Overlap in assemblies

	**Metavelvet, *k *** **= 39**	**Metavelvet, *k *** **= 51**	**Oases, *k *** **= 27–35**	**Oases, *k *** **= 39–45**	**Oases, *k *** **= 51–53**	**Trinity default**
All single-end contigs						
Metavelvet, *k* = 27	85.50%	69.60%	76.80%	76.20%	59.70%	98.00%
Metavelvet, *k* = 39		84.70%	64.50%	65.00%	71.20%	96.80%
Metavelvet, *k* = 51			64.30%	68.00%	48.10%	96.60%
Oases, *k* = 27–35				85.60%	68.10%	95.40%
Oases, *k* = 39–45					91.00%	97.30%
Oases, *k* = 51–53						97.60%
BLAST score >50						
Metavelvet, *k* = 27	87.20%	79.60%	72.80%	78.40%	74.50%	97.00%
Metavelvet, *k* = 39		89.80%	65.50%	64.70%	82.30%	96.50%
Metavelvet, *k* = 51			72.90%	69.50%	48.20%	97.10%
Oases, *k* = 27–35				92.60%	85.70%	88.00%
Oases, *k* = 39–45					92.00%	92.70%
Oases, *k* = 51–53						92.60%

Single-end assemblies constructed from 516,881 single-end reads of putative bacterial mRNA origin obtained from a non-obese diabetic (NOD) mouse cecal sample were evaluated on the uniqueness of the reads incorporated into contigs. Figures indicate the percentage of reads of the smaller dataset that are incorporated into contigs in both datasets.

### Comparison of assembly algorithms on paired-end sequence data

In the previous sections, we examined the performance of assemblers applied to single-end reads. To examine how paired-end sequencing for metatranscriptomic studies could augment assembly and annotation, we generated a set of 29.8 million 76-bp paired-end reads (average insert size 273 bp) from the same rRNA-depleted samples used to generate the single-end reads [12] (Additional file 1). We observed a high degree of consistency between single- and paired-end reads in terms of: 1) the relative proportion of sample represented in the entire dataset, 2) reads assigned to ribosomal transcripts for each sample, and 3) reads assigned to mouse host transcripts for each sample (Additional file 4). In contrast, the proportion of reads assigned to putative bacterial mRNA transcripts was lower for the paired-end reads and was associated with a rise in frequency of reads filtered on the basis of vector contamination. This is likely related to the processing step that discarded both reads in a pair even if only one member contained significant vector contamination. Interestingly, we note discrepancies between single and paired read data in the phylogenetic distribution of reads (Additional file 4). However, overall there is reasonable correlation between samples (*r*^2^ values from 0.75 to 0.99). For both ends of a read, this correlation was even higher (*r*^2^ from 0.95 to 1), suggesting that differences arise from biases introduced in sample preparation prior to sequencing, rather than bioinformatics processing.

Next, we evaluated potential performance gains through the use of paired-end reads. The paired-end reads allowed an evaluation of a dedicated metatranscriptomic assembly algorithm, IDBA-MT [20], as it is compatible only with paired-end reads. Compared to the single-end read data, we were able to assemble more contigs (Figure 3), potentially reflecting the greater number of the paired-end reads (553,115 pairs of reads vs. 516,881 single reads). For example, after dividing the paired ends into two separate datasets of 553,115 reads, Trinity generated 51,244 and 52,549 contigs, respectively. These data contrasted with 48,469 contigs for an assembly based on the 516,881 single-end reads and 58,361 contigs for an assembly based on combining both ends of the 553,115 pairs. However, compared to the single-end assembly, we were able to assemble a higher proportion of reads into contigs, resulting in a concomitant gain in the proportion of reads assembled into annotatable contigs (from 50.3% to 64.4% for the Trinity-based assembly; Figure 3). Greater performance gains were observed for the Metavelvet and Oases algorithms. Again, imposing a 150-bp contig size cutoff on the Trinity assembly resulted in a proportion of mapped reads comparable to Oases and Metavelvet despite a smaller number of overall contigs (Figure 3). In contrast, IDBA-MT did not perform as well as the other methods in either contigs produced or reads mapped to annotatable contigs. These findings are contrary to a previous report that Trinity generated only ~5% more contigs than IDBA-MT [20]. This discrepancy might arise because this latter study combined reads from all 12 paired-end mouse samples resulting in potential coverage saturation that may have produced a convergence in the number of contigs. However, assemblies based on the entire set of sequences from all 12 samples also resulted in a greater number of contigs using Trinity (127,511 contigs) compared to IDBA-MT (16,582 contigs). Instead, this discrepancy likely arises from the use of a default parameter in the Trinity software, which reports contigs only in excess of 200 bp (resulting in 10,823 contigs). To avoid differential biases between the assemblers, we reduced this stipulation to 51 bp (the minimum allowed read length after filtering) in our analyses. The overlap profile of the paired-end read assemblies was similar to the single-end data, again suggesting little benefit in combining results from different assemblers (Additional file 5).

### Database evaluation of transcript reconstruction accuracy across assemblers

**Figure 4:**
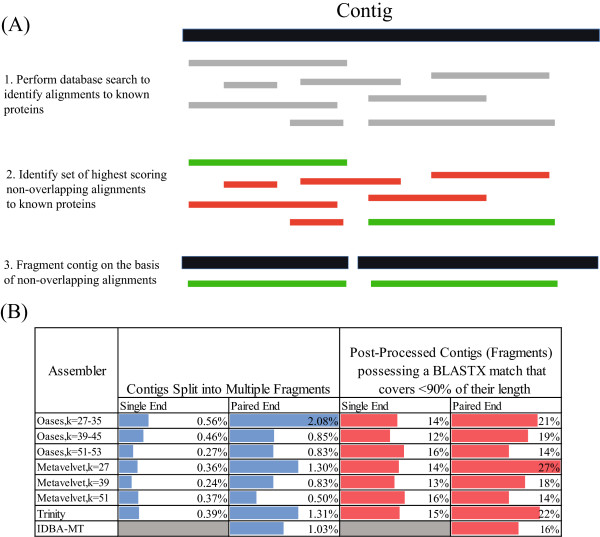
**Identification and evaluation of misassembled contigs. (A)** Strategy used to identify misassembled contigs with the potential to align to multiple bacterial proteins. First, we perform a database search to identify proteins aligning to the contig (1). Next, iterating from the start of the contig, we identify the set of highest scoring non-overlapping alignments (2). Based on these, the contig is subsequently fragmented (3). **(B)** Incidence of misassembles, as defined from the heuristic presented in (A), generated from both the single-end and paired-end read datasets generated from the NOD503CecMN sample (left panel). Also shown is the proportion of intact contigs and fragments which align <90% of their length to a known protein (right panel).

A major challenge for the assembly of metatranscriptomic datasets is the generation of contigs derived from two or more distinct genes either between unrelated transcripts sharing a region of high sequence similarity, gene fusions or as a result of polycistronic microbial transcripts. Sequence assembly improves the ability to annotate reads, but the generation of these misassembled or multifunctional transcripts may confound interpretation of resultant expression profiles. In the absence of a comprehensive set of reference genomes, we developed a heuristic algorithm to identify such contigs based on BLAST sequence similarity matches [22]. We then split these transcripts into fragments corresponding to a single putative gene (Figure 4). In brief, we ran a BLASTX search for a contig against the non-redundant protein database. We then identified the sequence match with the highest alignment score (bit score ≥50) by iterating over the entire contig sequence. A base that was already covered by a previous alignment was ignored. Subsequently, the contigs were split into discrete fragments if we identified two or more non-contiguous sequence alignments. We then noted all contigs composed of multiple fragments. This approach has two limitations. First, it relies on correctly annotated entries in the non-redundant protein database that are not the result of misassembly. Second, we assume that a misassembly does not generate a contig with similarity to a known gene that was not present in the metatranscriptomic sample.

Applying this procedure to our assemblies, we found that those based on single-end reads contained a low incidence of contigs composed of multiple genes (from 0.56% to 0.24% of contigs for Oases with *k* = 27–35 and Metavelvet with *k* = 39, respectively; Figure 4). Perhaps surprisingly, assemblies based on paired-end reads contained a higher incidence of misassembled contigs (from 2.08% to 0.5% of contigs for Oases with *k* = 27–35 and Metavelvet with *k* = 51, respectively). Both Trinity- and IDBA-MT-based assemblies gave comparable outcomes (1.31% and 1.03% of contigs, respectively). This increase in misassembled contigs associated with the paired-end reads is likely related to the increased read coverage provided by the dataset, resulting in longer contigs. Whether these misassembled contigs arise from the reconstruction of polycistronic mRNAs or assembly errors remains to be resolved. In any event, it is clear that while the higher coverage of the paired-end reads improves both the number of contigs and number of reads assigned to an annotatable contig, it has not improved accuracy of contig assembly.

Given the low incidence of misassembled contigs independent of assembler as determined by the above heuristic, we next looked for further evidence of potential misassembly through the identification of contigs with only partial matches to known proteins. For contigs (and fragments) possessing significant sequence similarity to a single known protein (as defined by a BLASTX match with a bit score cutoff of 50), we identified those for which the alignment with the protein covered less than 90% of the length of the contig or fragment. Such contigs or fragments may indicate either a gene fusion event which could not be resolved into discrete regions through the initial heuristic, a misassembly involving reads from two or more potentially related transcripts (e.g., members of the same gene family) that result in the generation of a hybrid transcript, or simply a novel gene that has yet to be captured by the non-redundant protein database. This latter type of sequence may be considered as a false-positive misassembly. Due to high rates of sequence divergence in microbiome samples, we expect such events to be a significant source of false-positive misassemblies. For example, a previous study suggests that ~8%–10% of genes associated with a newly sequenced genome are novel (i.e., lack sequence similarity to any known gene) [23]. Accordingly, this second heuristic yielded a higher estimate of potential misassemblies for both single- and paired-end datasets (13%–16% vs. 14%–27%, respectively; Figure 4). Interestingly, we note that increasing the *k* parameter in both Metavelvet and Oases increased reconstruction accuracy through both metrics.

While the incidence of contigs possessing non-overlapping alignments with two or more proteins was relatively low, we nonetheless propose the implementation of a post-assembly processing step such as that outlined above to convert contigs into discrete fragments associated with distinct sequence alignments. Note also that such a processing step also has the advantage of separating individual ORFs from assemblies of polycistronic mRNA moieties.

### Assembly of simulated metatranscriptomic datasets reveals transcript accuracy

**Figure 5:**
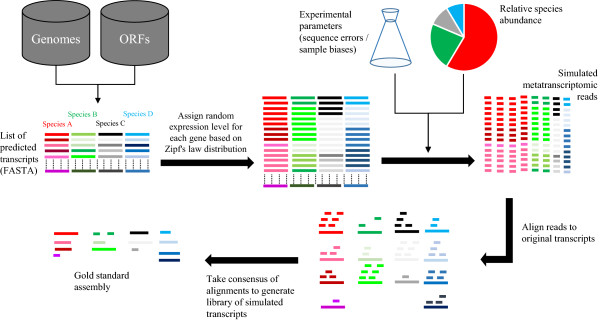
**Overview of metatranscriptome simulation pipeline based on FluxSimulator.** For each species considered, the genome sequence and ORF annotation file is used to create a list of predicted transcripts for each species. FluxSimulator then assigns each gene a random expression value based on Zipf’s law to create a library of the mRNA molecules that are present in the sample. Given a list of experimental parameter input (sequence errors, sample bias, and relative species abundance), a set of simulated metatranscriptomic reads are generated based on the set of transcripts provided. A gold standard assembly is then generated by aligning reads to the original transcripts and obtaining consensus sequences from the resulting alignments.

In the absence of reference genomes, it is only possible to estimate the accuracy of assembled transcripts from the mouse metatranscriptomic samples. To further inform on assembly accuracy, we therefore generated two simulated metatranscriptomic datasets of increasing complexity using a modified version of the RNA-Seq simulator, FluxSimulator [24] (Figure 5). FluxSimulator was originally developed to generate simulated reads from a model transcriptome, taking into account inherent biases that arise from both sample preparation steps such as reverse transcription, fragmentation, adapter ligation, and PCR amplification, as well as read error profiles that approximate those obtained from the Illumina sequencing platform. Two simulated datasets were generated based on a metagenome analysis of a human stool sample (National Center for Biotechnology Information (NCBI) Biosample identifier: SRS011061 [26]). The first was composed of sequences generated from the 73 most abundant species within the same sample for which a reference genome is available. The second was composed of sequences generated from the ten most abundant species associated with the ten most abundant genera for which a reference genome was available. Maintaining the proportion of reads from each species as the initial sample, we generated 1.75 million 76-bp single-end reads as well as 1.75 million 76-bp paired-end reads for each dataset using a modified version of FluxSimulator [24]. Paired-end fragment length distributions were taken from experimental values derived for the mouse paired-end dataset.

The availability of a reference metatranscriptome also allows the evaluation of assembly performance through the DETONATE software package, a transcriptome assembly evaluator [27]. In brief, we used the combined transcriptome of the ten species dataset to train a probabilistic model which is subsequently used to evaluate the assemblies from the NOD mouse datasets. Our results suggest that Trinity gave the worst performance according to this measure (Additional file 6), consistent with the previously reported N50 values. However, while this might suggest that the Trinity assembly least captures the properties of a real metatranscriptome, depending on the annotation pipeline, favoring inclusiveness of assembly may be preferred if it does not introduce errors which confound the interpretation of the final assembly results.

**Figure 6:**
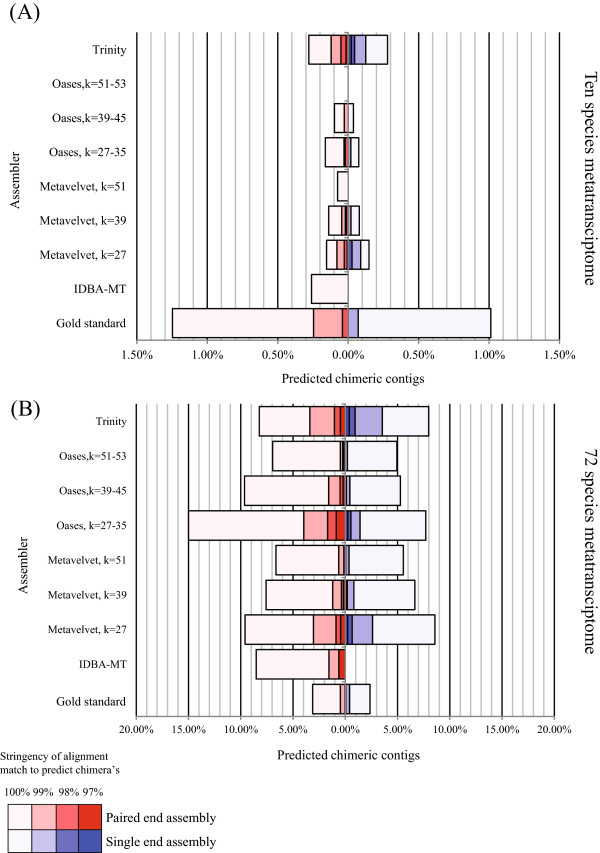
**Accuracy of simulated metatranscriptome assemblies.** For each simulated dataset, the accuracy of the reconstructed transcripts is evaluated based on their matches to the original set of transcripts used to generate the datasets. **(A)** Ten species dataset. **(B)** 72 species dataset. Shown is the percentage of contigs in each assembly which contain a region of at least one read length (76 bp) which does not align to a transcript at a variety of sequence cutoffs (97%–100% sequence identity). The gold standard assembly indicates the number of predicted misassemblies that are the result of introduced sequence errors during generation of the simulated datasets. Note this is higher for the ten species dataset as it includes a larger number of contigs than are generated by the assemblers (see text).

To examine the propensity of the four short-read assemblers to introduce misassemblies in more detail, we applied the assemblers to each simulated dataset and subsequently aligned resultant assemblies to the original reference genomes using BLAT [28]. As we are interested in investigating the incidence of large-scale assembly errors that may adversely impact functional annotation, misassembled contigs were defined as those in which greater than one read length (76 nucleotides) was unmatched in the highest scoring alignment to the reference genomes. To understand how sequencing error and depth could impact the number of false positives found by our metric, we also generated a ‘gold standard’ assembly by aligning simulated reads to the reference genomes and collating the resulting consensus sequences. For the ten species simulated dataset, we identified far fewer (<0.3%) misassembled contigs, compared with the NOD mouse datasets (Figure 6). Counterintuitively, we also identified fewer misassembled contigs than the gold standard assembly. However, this reduction in assembly errors for the *de novo* assemblers is likely related to the more limited number of contigs constructed (contigs assembled from reads with sequence errors in low coverage regions are likely to be rejected). This is reflected in the total number of contigs, reads assigned to contigs, and percentage of the metatranscriptome covered by each assembly (Additional file 6). Focusing on the 72 species simulated dataset, however, we identified many more misassembled transcripts (up to 15% for the simulated paired-end data assembled with Oases with *k* = 27–35; Figure 6). Given the high stringency used in matching contigs to the reference genomes, we next investigated the impact of match stringency on the prediction of large-scale misassembly events. Decreasing the percent identity match required for misassembly detection from 100% reduced the incidence to <5% and <1% for 99% identity and 98% identity, respectively. This suggests that most assembly errors in our simulation arise from sequence errors accumulated in the contigs rather than large-scale fusions of unrelated genes sharing a single region of similarity. Furthermore, given their poorer performance on the 72 species dataset relative to the gold standard assembly, these results demonstrate that datasets of increased complexity can result in assembly errors that are not simply the result of errors introduced during the sequencing process.

## Conclusions

We have shown that assembly of metatranscriptomic reads considerably improves short-read annotation. While only 15.5% of single-end reads obtained from the large intestine could be confidently assigned function, this percentage increases to 50.3 after assembly with Trinity. Furthermore, the number of contigs resulting from the fusion of two unrelated genes during the assembly process was rare in simple (8–10 species) experimental and simulated datasets. In a more complex simulated dataset composed of sequences from 72 species, there were many more assembly errors than expected by the sequence read quality. However, such errors appeared confined to relatively minor sequence variants rather than the merging of two unrelated genes that share a region of sequence similarity. While Trinity did not assemble the most accurate contigs, it significantly outperformed the other three assemblers in terms of the number of reads that could be aligned to a contig with known function. Future work will focus on improving the accuracy of reconstructed contigs in complex metatranscriptomic samples by first grouping reads into taxonomically defined bins, thereby reducing sample complexity prior to assembly. This algorithmic development can be expected to reduce assembly errors that arise from the merging of homologous transcripts from different species and subsequently improve taxonomic assignment and functional annotation of assembled contigs. Furthermore, while the focus of this study was on metatranscriptome assembly as well as the types of assembly errors that could impact downstream functional analyses, future work could focus on a systematic analysis of types of potential misassemblies and how assembler parameters may be optimized to differentiate between gene fusions, transcripts cotranscribed in operons, and genuine misassemblies.

## Methods

### Source and processing of sequencing reads

Single-end sequence reads from a previously published mouse gut metatranscriptome study were obtained from the Sequence Repository Archive (SRA051354) [29]. This dataset includes 12 samples generated from two different body sites, four different mice using a variety of different purification procedures described elsewhere [12]. Paired-end sequences were generated from the same barcoded libraries used to generate the single-end reads following standard Illumina protocols. Sequencing was performed with the Illumina Genome Analyzer II (GaII) platform at the Center for Advanced Genomics (TCAG - Hospital for Sick Children). After deconvolution of the barcodes used for multiplexing, 29,780,781 pairs of 76-bp reads were generated on a single flow cell. This paired-end data set, supporting the results of this article, is available from the Sequence Repository Archive (SRA051354 - http://www.ncbi.nlm.nih.gov/sra/?term=SRA051354) [29].

Compared with the previous publication, we applied a stricter protocol for removal of adaptor contaminants to optimize assembly performance; reads with adaptor or partial adaptor sequences at their ends may interfere with extension of transcripts during assembly. Adaptor sequences were identified using Cross_match (http://www.phrap.org) to search a database of Illumina adaptor sequences. We subsequently ran a more stringent screen focusing on the specific adaptors: AGATCGGAAGAGCACACGTCTGAACTCCAG and AGATCGGAAGAGCGTCGTGTAGGGAAAGA (minmatch 10, minscore 5). Poor-quality bases were removed by iterating a 5-nt window across the 5′ and 3′ ends of each sequence and removing nucleotides in windows with a mean quality score less than 20; iteration was stopped when the mean quality score was greater than 20. After trimming, reads less than 50 bp in length were discarded; for paired-end reads, if either read of a pair was less than 50 bp in length, then both reads were discarded. Putative rRNA reads were identified through BLAT [28] sequence similarity searches (bit score ≥50) against an in-house database of rRNA sequences [12]. Again, for paired-end reads, if either read of a pair matched a ribosomal sequence, both reads were annotated as being of rRNA in origin. Putative mouse transcript sequences were identified through BLAT sequence similarity searches (bit score ≥50) against a database of mouse genome and transcriptome sequences obtained from ENSEMBL release 67 [30]. Depending on sample, 3%–29% of the reads could be annotated as being of putative bacterial mRNA origin (Additional file 1). Phylogenetic annotations were performed by running BLASTX sequence similarity searches against the non-redundant protein database [31], using the highest scoring alignment (bit score >50). Resulting species were assigned to larger taxonomic groups with reference to the National Center for Biotechnology Information (NCBI) taxonomy tree [32].

### Generation of simulated metatranscriptomic datasets

Simulated metatranscriptomic datasets were generated based on sequence abundance data previously generated from the stool of a female participant of the Human Microbiome Project (HMP) (Biosample identifier: SRS011061 [26]). From an original list of 180 species, 73 were associated with a reference genome available from the Human Microbiome Reference Genome database (HMREFG [33]; Additional file 7). For each of these 72 species, annotation files were converted from GenBank format to gtf format for use in FluxSimulator [24] using a custom script. A total of 1.75 million 76-bp single-end and paired-end reads were generated with the proportion of each of the 72 species obtained with reference to the HMP sample. To generate a less complex dataset consisting of ten species, a single species representative was selected from each of the ten most abundant genera identified in the HMP sample. Again, 1.75 million single-end and paired-end reads were generated with the proportion of each of the ten species obtained with reference to the HMP sample. To generate a gold standard assembly for the simulated datasets that takes into account read errors introduced by FluxSimulator, we used a parallelised version of BLAT (https://code.google.com/p/pblat/) to align simulated reads to the set of reference genomes originally used to generate the reads. Since FluxSimulator includes sequence upstream of start codons in the generation of simulated transcripts, it can occasionally result in the generation of reads representing a fusion of two neighboring genes. For the purposes of defining misassemblies, these were ignored.

### Sequence assembly and mapping reads back to contigs

For Trinity [19], we used the following parameters: fastq assembly, 16 CPUs for Inchworm and Butterfly, a maximum heap size of 12 GB, and an insert distance of 270 bp for the paired-end assemblies. Only contigs in excess of 50 bp were reported. For Oases [18], we used version 2.0.8 and varied the minimum and maximum *k*-mer parameters with values listed in the text. Insert length was defined as 270 bp for the paired-end assemblies. For Metavelvet [17], we used version 1.2.01 coupled with Velvet [34] version 1.2.07. Velveth was initially run on the fastq files, using the -short parameter for the single-end reads and the -shortPaired parameter for the paired-end reads. Values for minimum and maximum *k*-mer parameters are listed in the text. Subsequently, velvetg was run using the -exp_cov auto parameter for both single- and paired-end reads. The ins_length parameter was set to 270 for the paired-end reads. Finally, meta-velvetg was run setting the -ins_length parameter to 270 for the paired assemblies. IDBA-MT [20] version 1.0 was run on contigs initially generated using IDBA-UD [35] version 1.0.9 on paired-end reads using default parameters with an insert length of 270. To map reads to contigs, we applied BWA [36] with default parameters. To calculate the overlap between reads mapping to different contig sets, we calculated the intersection of the reads mapping to the two different assemblies divided by the size of smallest set of mapped reads.

## Competing interests

The authors declare that they have no competing interests.

## Authors’ contributions

AC performed sequence processing, algorithm development, assembly, and analyses. JM and JD generated paired-end sequence data. AC, JD, and JP drafted the manuscript. The study was designed and conceived by AC and JP. All authors read and approved the final manuscript.

## Supplementary Material

Additional file 1 **Sequence yields for 12 NOD mouse sample preparations.** Table showing number and breakdown of sequence reads generated from single and paired-end sequencing runs. Click here for file

Additional file 2 **Number of reads and proportion with BLASTX matches for 12 samples of single- and paired-end reads derived from the large intestine of non-obese diabetic mice.** Graph shows the proportion of reads which can be annotated through sequence similarity searches. Click here for file

Additional file 3 **Overlap in single-end assemblies.** Single-end assemblies constructed from 516,881 single-end reads of putative bacterial mRNA origin obtained from a non-obese diabetic (NOD) mouse cecal sample were evaluated on the uniqueness of the reads incorporated into contigs. The size of circles and overlap areas is approximately proportional to the reads incorporated into each assembly and the read profile overlaps for a) Oases, b) Metavelvet, and c) Trinity compared to Oases and Metavelvet with the lowest *k* parameters. Click here for file

Additional file 4 **Comparisons of the performance of single- and paired-end sequence reads generated from the large intestine of non-obese diabetic mice.** Graphs show consistency of single- and paired-end datasets in terms of rRNA, mouse RNA, and bacterial mRNA representation, as well as phylogenetic breakdown of annotatable reads. Click here for file

Additional file 5 **Overlap in assemblies of paired-end reads.** As for Table 1, this table shows the overlap of reads incorporated into the various assemblies. Click here for file

Additional file 6 **Statistics of simulated metatranscriptome assemblies constructed from ten species.** Table showing performance of various assemblies on the simulated metatranscriptome dataset constructed from ten species. Click here for file

Additional file 7 List of species used to generate the simulated datasets. Click here for file
