# Supplementary material for: Comparison of assembly algorithms for improving rate of metatranscriptomic functional annotation
Source: Microbiome. 2014 Oct 28;2:39. doi: 10.1186/2049-2618-2-39 (PMC4236897; doi:10.1186/2049-2618-2-39)
Supplement: Additional file 1 — Sequence yields for 12 NOD mouse sample preparations. Table showing number and breakdown of sequence reads generated from single and paired-end sequencing runs. [file 2049-2618-2-39-S1.docx]

| **Single end reads** | | | | | | |
| --- | --- | --- | --- | --- | --- | --- |
| **Sample Name** | **Sequenced Reads** | **Vector and Low Quality** | **Ribosomal RNA** | **Mouse Genome and Transcriptome** | **Putative mRNA** | |
|  |  |  |  |  | **Total Reads** | **% of all reads** |
| **NOD501CecQN** | 1463097 | 587076 | 771458 | 10606 | 93957 | 6% |
| **NOD501CecQY** | 2241568 | 1061645 | 439119 | 90745 | 650059 | 29% |
| **NOD501ColQN** | 1334211 | 75474 | 1143119 | 5784 | 109834 | 8% |
| **NOD502CecQN** | 2178329 | 715760 | 1255566 | 19974 | 187029 | 9% |
| **NOD502CecQY** | 1501515 | 831498 | 454340 | 30882 | 184795 | 12% |
| **NOD502ColQN** | 1149057 | 160065 | 807061 | 88727 | 93204 | 8% |
| **NOD503CecMN** | 2139350 | 485349 | 615920 | 521200 | 516881 | 24% |
| **NOD503CecQN** | 1626214 | 440574 | 1029822 | 13202 | 142616 | 9% |
| **NOD504CecMN** | 2614525 | 388107 | 575483 | 1243606 | 407329 | 16% |
| **NOD504CecQN** | 1986083 | 537543 | 1231790 | 24736 | 192014 | 10% |
| **NOD504CecQY** | 2770382 | 1211800 | 1051429 | 54205 | 452948 | 16% |
| **NOD504ColQN** | 1710072 | 341164 | 738720 | 369238 | 260950 | 15% |

| **Paired end reads (number of paired sequences)** | | | | | | | |
| --- | --- | --- | --- | --- | --- | --- | --- |
| **Sample Name** |  | **Pairs of Sequenced Reads** | **Vector and Low Quality** | **Ribosomal RNA** | **Mouse Genome and Transcriptome** | **Putative mRNA** | |
|  | **Insert size** |  |  |  |  | **Total pairs** | **% of all pairs** |
| **NOD501CecQN** | 260 | 2246294 | 1099609 | 1074624 | 7507 | 64554 | 3% |
| **NOD501CecQY** | 270 | 2931351 | 1701086 | 497434 | 65221 | 667610 | 23% |
| **NOD501ColQN** | 214 | 1370497 | 118209 | 1203067 | 3444 | 45777 | 3% |
| **NOD502CecQN** | 229 | 3135201 | 1290981 | 1708720 | 10702 | 124798 | 4% |
| **NOD502CecQY** | 348 | 2021696 | 1292560 | 535178 | 19306 | 174652 | 9% |
| **NOD502ColQN** | 273 | 1579372 | 266038 | 1113212 | 117592 | 82530 | 5% |
| **NOD503CecMN** | 280 | 3087505 | 947557 | 870936 | 715897 | 553115 | 18% |
| **NOD503CecQN** | 263 | 1720937 | 547169 | 1102327 | 6136 | 65305 | 4% |
| **NOD504CecMN** | 259 | 2676824 | 545971 | 570524 | 1250281 | 310048 | 12% |
| **NOD504CecQN** | 307 | 2522460 | 824794 | 1567844 | 15711 | 114111 | 5% |
| **NOD504CecQY** | 283 | 4142841 | 2380608 | 1312773 | 46795 | 402665 | 10% |
| **NOD504ColQN** | 295 | 2345803 | 621637 | 984793 | 486934 | 252439 | 11% |

**Sequence yields for 12 NOD Mouse sample preparations**.

Sequences were generated using the Illumina GaIIx platform for 12 cecal and colon samples derived from four different NOD mice (labelled 501, 502, 503 and 504). Two runs were performed in which all twelve samples were multiplexed on a single Illumina sequencing lane. The first was a single end run which generated 22.7 million sequences. The second was a paired end run which generated 29.8 million pairs of reads. Samples were further defined based on location (Cec = cecum / Col = colon; Q = RNA purification through RNeasy kit – Qiagen Inc / M = RNA purification through mirVana kit – Invitrogen Inc.; N / Y indicates application of Ribominus Transcriptome Isolation Kit – Qiagen Inc.)
