# Supplementary material for: Comparison of assembly algorithms for improving rate of metatranscriptomic functional annotation
Source: Microbiome. 2014 Oct 28;2:39. doi: 10.1186/2049-2618-2-39 (PMC4236897; doi:10.1186/2049-2618-2-39)
Supplement: Additional file 2 — Number of reads and proportion with BLASTX matches for 12 samples of single- and paired-end reads derived from the large intestine of non-obese diabetic mice. Graph shows the proportion of reads which can be annotated through sequence similarity searches. [file 2049-2618-2-39-S2.docx]

**Number of Reads and Proportion with BLASTX matches for twelve samples of single and paired end reads derived from the large intestine of non-obese diabetic mice.**

Sample identifiers are provided in additional file 1. Paired end sequences are identified with the suffix’s: ‘left’ and ‘right’. While the number of reads varies considerably between sample preparations, the proportion of filtered reads with a database match to a prokaryotic peptide (as determined with a BLAST bit score cutoff of 50) is consistently below 20%, suggesting ability to annotate reads is not biased by sample preparation.
