# Supplementary figures and images for: Comparison of assembly algorithms for improving rate of metatranscriptomic functional annotation
Source: Microbiome. 2014 Oct 28;2:39. doi: 10.1186/2049-2618-2-39 (PMC4236897; doi:10.1186/2049-2618-2-39)

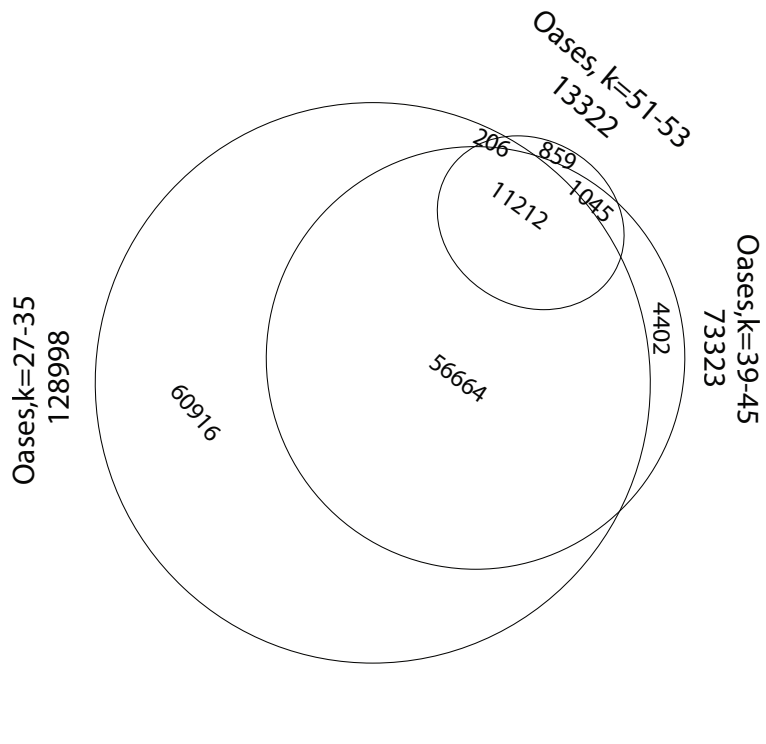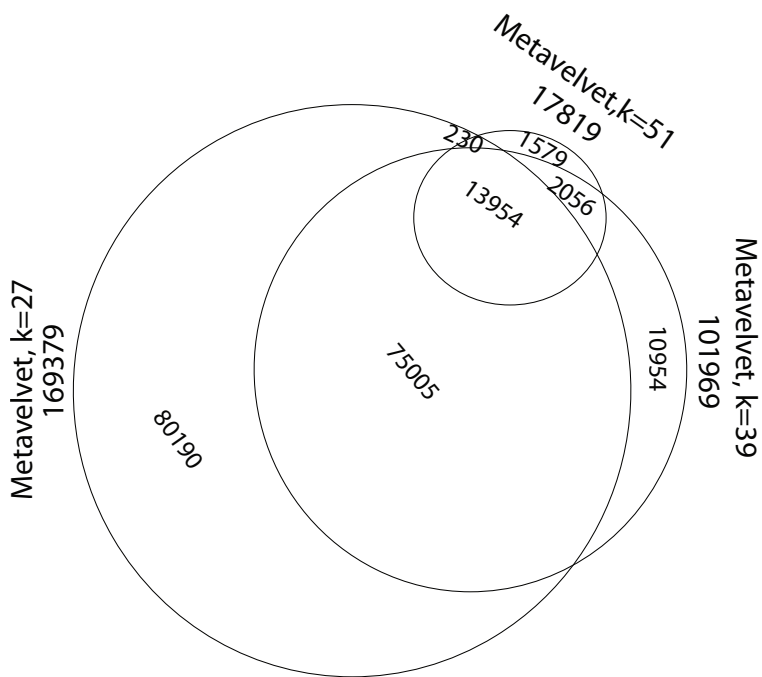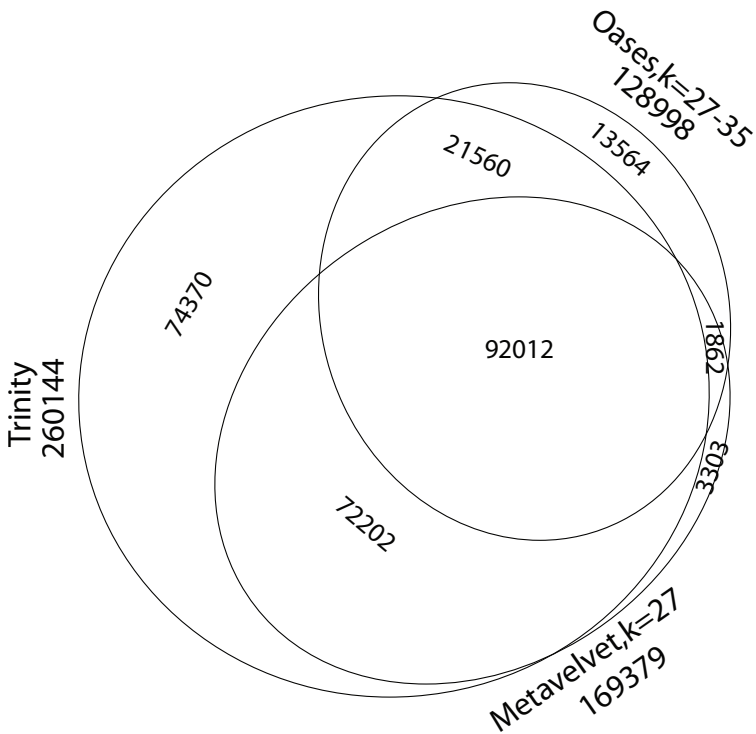

Supplement: Additional file 3 — Overlap in single-end assemblies. Single-end assemblies constructed from 516,881 single-end reads of putative bacterial mRNA origin obtained from a non-obese diabetic (NOD) mouse cecal sample were evaluated on the uniqueness of the reads incorporated into contigs. The size of circles and overlap areas is approximately proportional to the reads incorporated into each assembly and the read profile overlaps for a) Oases, b) Metavelvet, and c) Trinity compared to Oases and Metavelvet with the lowest k parameters. [file 2049-2618-2-39-S3.pdf]

(A)

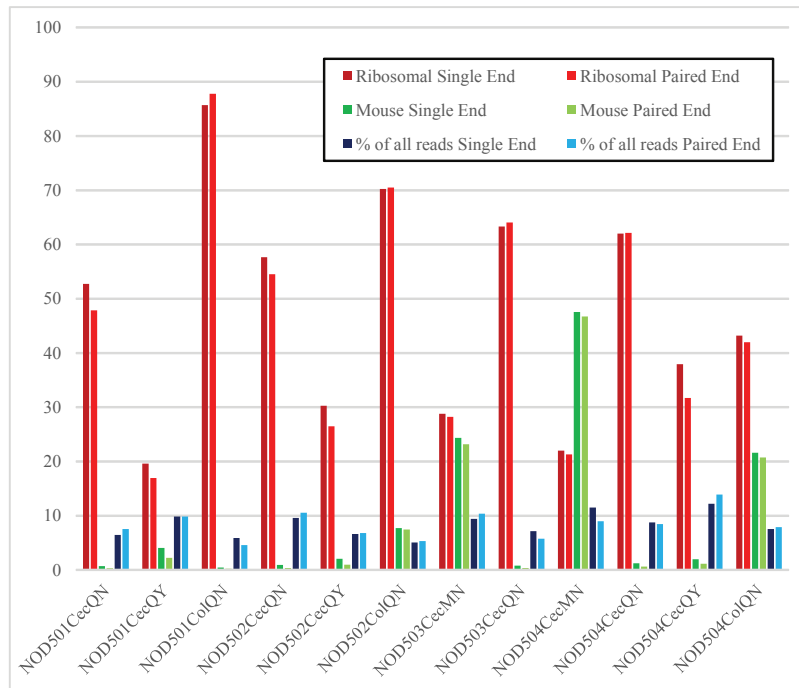

(B)

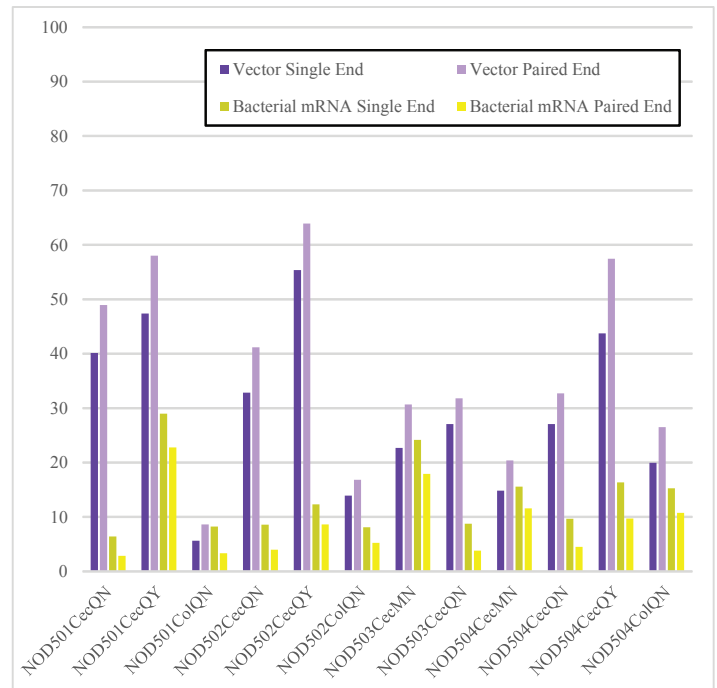

(C)

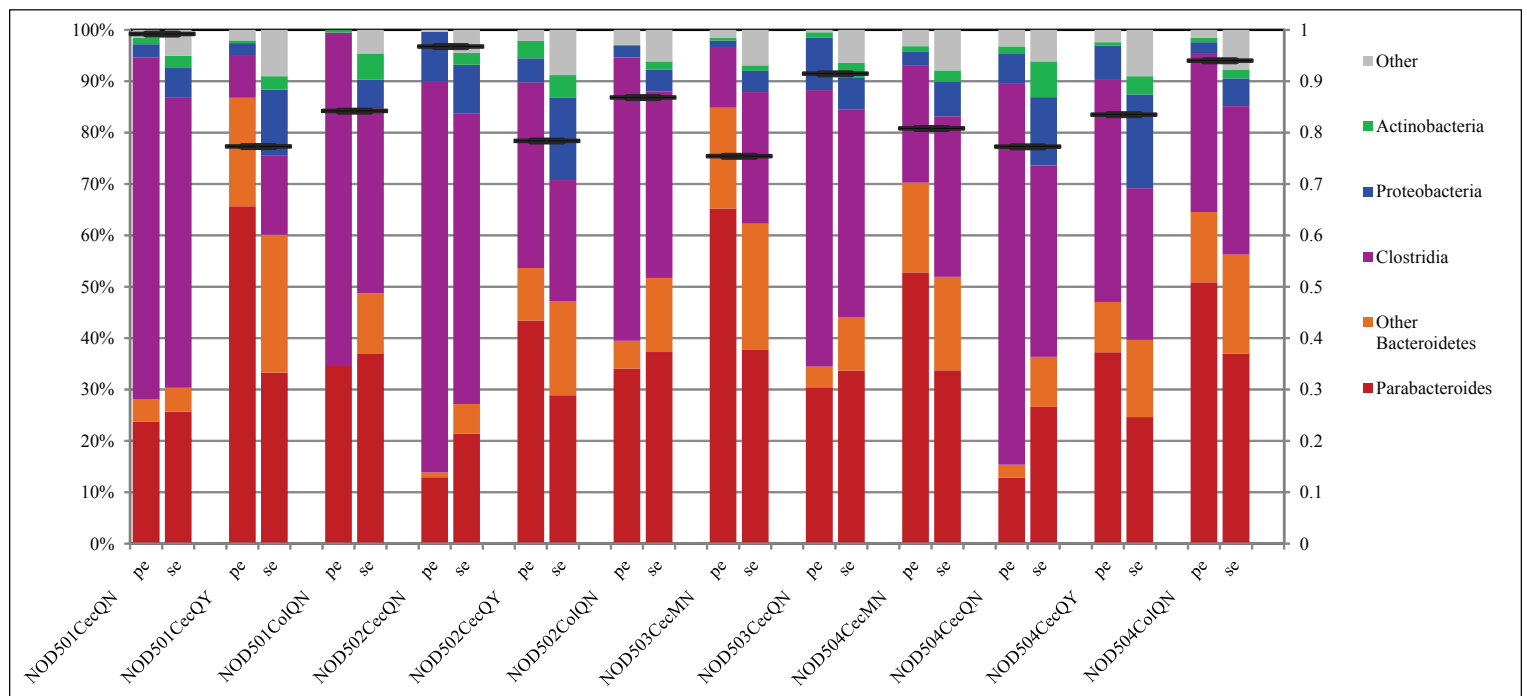

Supplement: Additional file 4 — Comparisons of the performance of single- and paired-end sequence reads generated from the large intestine of non-obese diabetic mice. Graphs show consistency of single- and paired-end datasets in terms of rRNA, mouse RNA, and bacterial mRNA representation, as well as phylogenetic breakdown of annotatable reads. [file 2049-2618-2-39-S4.pdf]
