# Supplementary material for: Comparison of assembly algorithms for improving rate of metatranscriptomic functional annotation
Source: Microbiome. 2014 Oct 28;2:39. doi: 10.1186/2049-2618-2-39 (PMC4236897; doi:10.1186/2049-2618-2-39)
Supplement: Additional file 5 — Overlap in assemblies of paired-end reads. As for Table 1, this table shows the overlap of reads incorporated into the various assemblies. [file 2049-2618-2-39-S5.docx]

| **All Paired End Contigs** | **Metavelvet k=27** | **Metavelvet k=39** | **Metavelvet k=51** | **Oases\n k=27-35** | **Oases\n k=39-45** | **Oases\n k=51-53** | **Trinity Default** |
| --- | --- | --- | --- | --- | --- | --- | --- |
| **IDBA-MT** | 81.84% | 80.20% | 57.61% | 96.06% | 85.73% | 63.22% | 97.93% |
| **Metavelvet,k=27** |  | 90.00% | 83.70% | 90.50% | 87.00% | 83.90% | 97.90% |
| **Metavelvet,k=39** |  |  | 89.40% | 96.60% | 93.40% | 89.20% | 97.40% |
| **Metavelvet,k=51** |  |  |  | 95.00% | 92.00% | 99.00% | 97.40% |
| **Oases,k=27-35** |  |  |  |  | 96.30% | 95.40% | 96.80% |
| **Oases,k=39-45** |  |  |  |  |  | 93.50% | 97.80% |
| **Oases,k=51-53** |  |  |  |  |  |  | 97.70% |
| **Blast score >50** | **Metavelvet k=27** | **Metavelvet k=39** | **Metavelvet k=51** | **Oases\n k=27-35** | **Oases\n k=39-45** | **Oases\n k=51-53** | **Trinity Default** |
| **IDBA-MT** | 84.86% | 82.31% | 55.31% | 96.16% | 85.10% | 60.36% | 91.24% |
| **Metavelvet,k=27** |  | 90.00% | 88.40% | 95.50% | 88.40% | 89.50% | 96.80% |
| **Metavelvet,k=39** |  |  | 93.80% | 96.90% | 94.10% | 94.20% | 96.70% |
| **Metavelvet,k=51** |  |  |  | 96.00% | 93.20% | 98.30% | 96.50% |
| **Oases,k=27-35** |  |  |  |  | 97.00% | 96.00% | 89.60% |
| **Oases,k=39-45** |  |  |  |  |  | 94.50% | 94.90% |
| **Oases,k=51-53** |  |  |  |  |  |  | 97.40% |

**Additional File 4. Table showing overlap in assemblies of paired end reads**

Paired-end assemblies constructed from 553,115 pairs of reads of putative bacterial mRNA origin obtained from a non-obese diabetic (NOD) mouse cecal sample, were evaluated on the uniqueness of the reads incorporated into contigs. Figures indicate the percentage of reads of the smaller dataset that are incorporated into contigs in both datasets.
