# Supplementary material for: Comparison of assembly algorithms for improving rate of metatranscriptomic functional annotation
Source: Microbiome. 2014 Oct 28;2:39. doi: 10.1186/2049-2618-2-39 (PMC4236897; doi:10.1186/2049-2618-2-39)
Supplement: Additional file 6 — Statistics of simulated metatranscriptome assemblies constructed from ten species. Table showing performance of various assemblies on the simulated metatranscriptome dataset constructed from ten species. [file 2049-2618-2-39-S6.docx]

**Additional File 5.**

**RSEM-eval score of various assemblies on a single and paired end metatranscriptomic data.** Using RSEM-EVAL from the DETONATE package, a score for each assembly as calculated which is an estimate of metatranscriptome reconstruction accuracy [[1](#_ENREF_1)]. In brief, a model transcriptome was generated from the 10 species simulation by creating a fasta file containing all annotated genes belonging to those species. Using the DETONATE package, estimateParams was run on this file to estimate the prior properties of a complete assembly of the sample. The assembly files for the single and paired end data were then prepared as described by the authors (with --no-polyA enabled), and using a read length of 50bp (as we sequenced 76bp but allowed removal of contaminating bases up until 50 were remaining) the scores were calculated for the single and paired end assemblies. A more negative score indicates better performance.

|  | **Single End Score** | **Paired End Score** |
| --- | --- | --- |
| **Trinity** | -26299734 | -47106393 |
| **Metavelvet,k=27** | -38511900 | -67225528 |
| **Metavelvet,k=39** | -43655736 | -76393293 |
| **Metavelvet,k=51** | -50629288 | -96723150 |
| **Oases,k=27-35** | -43015678 | -65194486 |
| **Oases,k=39-45** | -46152368 | -77832094 |
| **Oases,k=51-53** | -51046742 | -100732268 |
| **IDBA-MT** |  | -80745303 |

**Statistics of simulated metatranscriptome assemblies constructed from ten species.** Sequence assemblies were generated for the simulated metatranscriptomic datasets constructed from ten and 73 species. Shown here are statistics associated with the ten species dataset. Proportion of microbiome, indicates the % of the transcriptome associated with the ten species, that were covered by contigs assembled by the various methods. Proportion of reads mapping to a contig was obtained by applying BWA to align simulated reads to assembled contigs.

| assembler | Proportion of microbiome covered by contigs | Total contig bases | Number of contigs | Proportion of reads mapping to contig |
| --- | --- | --- | --- | --- |
| Gold Standard | 61.22% | 24878469 | 212636 | 100.0% |
| Metavelvet k=27 paired | 13.72% | 5574050 | 26478 | 41.5% |
| Metavelvet k=39 paired | 8.46% | 3438762 | 14991 | 30.5% |
| Metavelvet k=51 paired | 2.53% | 1030053 | 4682 | 16.3% |
| Oases k=27-35 paired | 17.66% | 7175267 | 24104 | 28.9% |
| Oases k=39-45 paired | 7.70% | 3130132 | 13421 | 21.4% |
| Oases k=51-53 paired | 1.81% | 734431 | 2986 | 12.3% |
| Trinity paired | 23.60% | 9592824 | 52362 | 70.4% |
| IDBA-MT | 6.47% | 2627504 | 11839 | 43.6% |
|  |  |  |  |  |
| Gold Standard | 41.13% | 16715845 | 165832 | 100.0% |
| Metavelvet k=27 single | 7.92% | 3217884 | 19827 | 26.8% |
| Metavelvet k=39 single | 3.29% | 1338626 | 8816 | 17.7% |
| Metavelvet k=51 single | 0.31% | 126838 | 715 | 8.3% |
| Oases k=27-35 single | 6.08% | 2470073 | 15180 | 15.9% |
| Oases k=39-45 paired | 2.56% | 1042205 | 6024 | 11.3% |
| Oases k=51-53 paired | 0.23% | 91903 | 453 | 5.9% |
| Trinity single | 14.81% | 6017828 | 44731 | 56.1% |

1. Li B, Fillmore N, Bai Y, Collins M, Thomson JA, Stewart R, Dewey C: **Evaluation of de novo transcriptome assemblies from RNA-Seq data**; 2014.
