# Supplementary material for: Comparison of assembly algorithms for improving rate of metatranscriptomic functional annotation
Source: Microbiome. 2014 Oct 28;2:39. doi: 10.1186/2049-2618-2-39 (PMC4236897; doi:10.1186/2049-2618-2-39)
Supplement: Additional file 7 — List of species used to generate the simulated datasets. [file 2049-2618-2-39-S7.docx]

**Additional File 6. List of species used to construct the simulated metatranscriptome datasets**

**73 species dataset**

Bacteroides sp. D2

Bacteroides uniformis ATCC 8492

Bacteroides sp. 2_2_4

Bacteroides cellulosilyticus DSM 14838

Bacteroides sp. 1_1_6

Clostridium hathewayi DSM 13479

Clostridium bolteae ATCC BAA-613

Bacteroides ovatus ATCC 8483

Clostridium asparagiforme DSM 15981

Bacteroides intestinalis DSM 17393

Bacteroides sp. D1

Bacteroides sp. 9_1_42FAA

Bacteroides fragilis 3_1_12

Bacteroides dorei 5_1_36/D4

Bacteroides dorei DSM 17855

Bacteroides sp. 4_3_47FAA

Bacteroides sp. 2_1_7

Bacteroides sp. 3_2_5

Bacteroides finegoldii DSM 17565

Parabacteroides johnsonii DSM 18315

Bacteroides caccae ATCC 43185

Parabacteroides merdae ATCC 43184

Bacteroides plebeius DSM 17135

Roseburia intestinalis L1-82

Bacteroides coprocola DSM 17136

Bacteroides eggerthii DSM 20697

Clostridium difficile NAP08

Roseburia inulinivorans DSM 16841

Bacteroides stercoris ATCC 43183

Clostridium hylemonae DSM 15053

Clostridium nexile DSM 1787

Bacteroides coprophilus DSM 18228

Clostridium sp. M62/1

Holdemania filiformis DSM 12042

Clostridium sp. 7_2_43FAA

Anaerotruncus colihominis DSM 17241

Ruminococcus obeum ATCC 29174

Clostridium scindens ATCC 35704

Anaerostipes caccae DSM 14662

Blautia hydrogenotrophica DSM 10507

Prevotella copri DSM 18205

Ruminococcus gnavus ATCC 29149

Clostridium methylpentosum DSM 5476

Eubacterium hallii DSM 3353

Clostridium leptum DSM 753

Coprococcus comes ATCC 27758

Subdoligranulum variabile DSM 15176

Clostridium ramosum DSM 1402

Dorea formicigenerans ATCC 27755

Clostridium sp. SS2/1

Faecalibacterium prausnitzii M21/2

Coprococcus eutactus ATCC 27759

Faecalibacterium prausnitzii A2-165

Blautia hansenii DSM 20583

Clostridium bartlettii DSM 16795

Clostridium sp. L2-50

Dorea longicatena DSM 13814

Eubacterium ventriosum ATCC 27560

Desulfovibrio piger ATCC 29098

Ruminococcus torques ATCC 27756

Ruminococcus lactaris ATCC 29176

Eubacterium siraeum DSM 15702

Catenibacterium mitsuokai DSM 15897

Mitsuokella multacida DSM 20544

Alistipes putredinis DSM 17216

Clostridium spiroforme DSM 1552

Butyrivibrio crossotus DSM 2876

Collinsella aerofaciens ATCC 25986

Clostridium hiranonis DSM 13275

Eubacterium biforme DSM 3989

Collinsella stercoris DSM 13279

Anaerofustis stercorihominis DSM 17244

Eubacterium dolichum DSM 3991

Collinsella intestinalis DSM 13280

Parvimonas micra ATCC 33270

**10 species dataset**

Bacteroides sp. D2

Clostridium hathewayi DSM 13479

Parabacteroides johnsonii DSM 18315

Roseburia intestinalis L1-82

Clostridium difficile NAP08

Holdemania filiformis DSM 12042

Anaerotruncus colihominis DSM 17241

Ruminococcus obeum ATCC 29174

Anaerostipes caccae DSM 14662

Blautia hydrogenotrophica DSM 10507
